# Structure and Electron-Transfer Pathway of the Human Methionine Sulfoxide Reductase MsrB3

**DOI:** 10.1089/ars.2020.8037

**Published:** 2020-08-27

**Authors:** Gabriel Javitt, Zhenbo Cao, Efrat Resnick, Ronen Gabizon, Neil J. Bulleid, Deborah Fass

**Affiliations:** ^1^ Department of Structural Biology, Weizmann Institute of Science, Rehovot, Israel.; ^2^ Institute of Molecular, Cellular and Systems Biology, CMVLS, University of Glasgow, Glasgow, United Kingdom.; ^3^ Department of Organic Chemistry, Weizmann Institute of Science, Rehovot, Israel.

**Keywords:** MsrB, disulfide, sulfenic acid, thiol-disulfide exchange, X-ray crystallography

## Abstract

***Aims:*** The post-translational oxidation of methionine to methionine sulfoxide (MetSO) is a reversible process, enabling the repair of oxidative damage to proteins and the use of sulfoxidation as a regulatory switch. MetSO reductases catalyze the stereospecific reduction of MetSO. One of the mammalian MetSO reductases, MsrB3, has a signal sequence for entry into the endoplasmic reticulum (ER). In the ER, MsrB3 is expected to encounter a distinct redox environment compared with its paralogs in the cytosol, nucleus, and mitochondria. We sought to determine the location and arrangement of MsrB3 redox-active cysteines, which may couple MsrB3 activity to other redox events in the ER.

***Results:*** We determined the human MsrB3 structure by using X-ray crystallography. The structure revealed that a disulfide bond near the protein amino terminus is distant in space from the active site. Nevertheless, biochemical assays showed that these amino-terminal cysteines are oxidized by the MsrB3 active site after its reaction with MetSO.

***Innovation:*** This study reveals a mechanism to shuttle oxidizing equivalents from the primary MsrB3 active site toward the enzyme surface, where they would be available for further dithiol-disulfide exchange reactions.

***Conclusion:*** Conformational changes must occur during the MsrB3 catalytic cycle to transfer oxidizing equivalents from the active site to the amino-terminal redox-active disulfide. The accessibility of this exposed disulfide may help couple MsrB3 activity to other dithiol-disulfide redox events in the secretory pathway.

## Introduction

The reversible modification of the sulfur-containing amino acid side chains cysteine and methionine is a common mechanism for regulation of protein function. Our knowledge of how cysteine side chains are oxidized and reduced is well advanced, but we are only beginning to appreciate how methionine side chains are reversibly modified (23). Methionine in proteins can be oxidized to methionine sulfoxide (MetSO) either nonspecifically by reactive oxygen species or as a specific, enzyme-catalyzed post-translational modification. On oxidation, the methionine sulfur atom becomes a new chiral center, producing methionine-*R*-sulfoxide or methionine-*S*-sulfoxide.

Two main classes of methionine sulfoxide reductase (Msr) enzymes catalyze MetSO reduction to restore the unmodified methionine: MsrA enzymes reduce the *S* epimer, and MsrB enzymes reduce the *R* epimer. As an example of regulation using this chemistry, enzymes of the Mical family stereoselectively oxidize methionine in actin, resulting in polymer disassembly (19), and cytosolic MsrB activity counteracts this process by reducing the actin MetSOs (28). MsrA is also involved in regulatory processes in the cell (33, 36). Though the catalytic activities of the two Msr classes are virtually identical, reactions on the epimers require a mirror-image positioning of functional groups in the enzyme active sites (32). MsrA and MsrB enzymes arose independently in evolution to act on the two forms of the substrate (49).

**Innovation**

This study describes the first high-quality structures of a mammalian MetSO reductase and reveals the spatial relationship between the resolving cysteines and the catalytic center. MetSO reductase activity of human MrsB3, which is targeted to the ER, was shown to be coupled to the generation of a disulfide bond involving the resolving cysteines. The accessibility of this disulfide and its appropriate geometry for further dithiol-disulfide exchange reactions provide an explanation for how MsrB3 activity may be integrated into the redox environment of the secretory pathway.

**FIG. 1.**
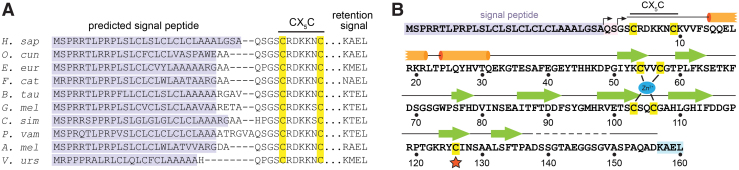
**MsrB3 sequence. (A)** Amino acid sequence alignments of the amino- and carboxy termini of representative mammalian MsrB3 orthologs (*Homo sapiens*, *Oryctolagus cuniculus*, *Erinaceus europaeus*, *Felis catus*, *Bos taurus*, *Globicephala melas*, *Ceratothenium simum*, *Pteropus vampyrus*, *Ailuropoda melanoleuca*, and *Vombatus ursinus*). Predicted signal sequences for ER targeting are highlighted in *violet*. *Dashes* indicate gaps in the alignment; *dots* indicate intervening sequence that is not shown. The CX_5_C motif, including the identities of the residues between the two cysteines, is highly conserved. KDEL-like ER retention signals are present at the carboxy termini. **(B)** Sequence of the precursor of ER-localized human MsrB3 with secondary structure annotations according to the structure reported herein. The signal peptide is highlighted in *violet*/*pink*, with two *arrows* indicating possible cleavage sites generating the mature protein. The first *arrow* indicates the site predicted by using SignalP (37). The second site and resulting numbering of the mature sequence are according to UniProt (45). The KDEL-like ER retention signal (KAEL in human MsrB3) is highlighted in *light blue*. Cysteine amino acids are highlighted in *yellow*. The *blue oval* represents a zinc ion coordinated by cysteine residues. The active-site cysteine is indicated by an *orange star*, and the resolving cysteines are indicated by an *overline* labeled CX_5_C. *Cylinders* indicate helices; *arrows* indicate β-strands. The *dashed* segment indicates a region for which no electron density was evident in the crystallographic maps. ER, endoplasmic reticulum; KAEL, Lys-Ala-Glu-Leu; KDEL, Lys-Asp-Glu-Leu; Msr, methionine sulfoxide reductase. Color images are available online.

Eukaryotes, eubacteria, and archaea encode Msr enzymes (49). Structural and biochemical insights have largely come from the study of prokaryotic Msr versions (32, 41, 44), but functional diversification of this enzyme family has occurred in multicellular organisms. Humans and other mammals have one MsrA and three MsrB paralogs, MsrB1, MsrB2, and MsrB3. MsrA is found in mitochondria and on the cytosolic face of endosomes (30). MsrB1 has an active-site selenocysteine and is found in the nucleus and cytosol (20). Both MsrB2 and MsrB3 contain active-site cysteines, but MsrB2 is present in mitochondria, and MsrB3, the focus of this article, has distinct splice variants targeted to mitochondria (MsrB3B isoform) or the endoplasmic reticulum (ER) (MsrB3A isoform, hereafter referred to as MsrB3) (20, 21). Notably, MsrB3 genes exhibiting ER signal peptides and ER retention coding sequences are present in genomes spanning mammalian phylogeny (Fig. 1A). Experimentally fusing the MsrB3 signal peptide and ER retention signal onto MsrA rerouted it to the ER, indicating that these sequences are functional for organelle targeting (24). Interestingly, an in-depth study of Lys-Asp-Glu-Leu-like ER retention signals revealed that MsrB3 remains localized to the ER even when truncated before its Lys-Ala-Glu-Leu (KAEL) sequence (42). The mechanism for ER retention of MsrB3 is not fully resolved, but the signal sequence is clearly capable of ER targeting.

Though the substrates of MsrB3 are as yet unknown, the enzyme has intriguing physiological associations. Human MsrB3 mutations are linked to non-syndromic deafness (5), and MsrB3 knockout correspondingly produces deafness in mice (47). MsrB3 expression levels affect the resistance of stem cells to oncogene-induced DNA damage (34, 39) and the resistance of cancer cells to apoptosis (26). MsrB3 expression was seen to change in patients with Alzheimer's disease (4), and MsrB3 single nucleotide polymorphisms are associated with hippocampal volume (18). In plants, an ER-localized MsrB protects against oxidative stress on exposure to cold (27). A key question is whether mammalian MsrB3 acts as a generic antioxidant enzyme or a specific regulatory factor in the ER, or both. Further study of adaptations of MsrB enzymes in mammals may provide insights into additional biochemical and functional capabilities.

In addition to potential substrates and physiological pathways, another aspect of MsrB3 activity that is likely affected by subcellular localization is the reactivation step. The need for reactivation to complete a catalytic cycle arises from the MsrB mechanism: A round of MetSO reduction produces a selenocysteine selenenic acid or a cysteine sulfenic acid, which must be reduced to regenerate a functional enzyme-active site. One common mechanism for reducing the active sites of MsrB enzymes is nucleophilic attack on the selenenic or sulfenic acid by the thiolate group of what is known as the “resolving” cysteine, forming a transient covalent link and liberating a water molecule. Reduction of the disulfide or selenium-sulfur bond, which can be accomplished by thioredoxin, then restores the active site (9). Despite the finding that aspects of normal ER function depend on the nicotinamide adenine dinucleotide phosphate/thioredoxin reductase/thioredoxin system (38), thioredoxin itself is not present in the ER and therefore cannot directly reactivate MsrB3 in that compartment. Recycling of MsrB3 may involve glutathione or ER-localized thioredoxin-fold proteins, such as members of the protein disulfide isomerase (PDI) family, and MsrB3 may have unique characteristics enabling recycling in the ER environment.

According to amino acid sequence alignments, the positions of the resolving cysteines differ among the human MsrB paralogs (22). MsrB1 is predicted to have a resolving cysteine in the vicinity of the selenocysteine in the three-dimensional structure of the enzyme (22) (see also protein data bank [PDB] code 2MAO, in which the region of the resolving cysteine is absent), similar to the arrangement found in MsrB enzymes of many microorganisms (*e.g.*, PDB code 3CXK). MsrB2 and MsrB3 lack a cysteine at the comparable position, but MsrB3 has two cysteines in a CX_5_C motif (in which X stands for a noncysteine amino acid) closely following the signal peptide (Fig. 1A, B). Either of these CX_5_C cysteines can catalyze regeneration of the active site in enzyme assays when thioredoxin is supplied *in vitro* as the reductant (10). However, how these cysteines function together in the MsrB3 catalytic cycle in the ER is an open question. The amino terminal region of MsrB3 shares no sequence homology with any MsrB enzyme with a known structure nor with MsrA. To reveal the locations of the additional cysteines in the protein and, more generally, to provide the first structure of an Msr enzyme that functions in the ER, we determined the human MsrB3 structure by using X-ray crystallography. Further biochemical analysis demonstrated that the redox state of the CX_5_C cysteines is coupled to activity at the enzyme active site.

## Results

### Structure determination and overall description

Human MsrB3, lacking the signal peptide and carboxy-terminal KAEL sequence (Fig. 1), was produced in *Escherichia coli* as a fusion with maltose binding protein (MBP). A tobacco etch virus (TEV) protease cleavage site enabled removal of the MBP. After cleavage and purification, MsrB3 was treated with dimethylamine-borane complex to methylate lysines as described (46), since attempts to crystallize the unmethylated protein were unsuccessful. The treated protein was crystallized by using the hanging drop vapor diffusion method, and crystals diffracting to 1.87 Å resolution (Table 1) were obtained. These crystals contained two MsrB3 molecules per asymmetric unit. The structure was solved by molecular replacement using a bacterial MsrB protein (PDB code 3CEZ) with high sequence identity to human MsrB3 (74 of 119 residues, or 62%) (6). Though the amino-terminal segment containing the CX_5_C cysteines did not exist in the search model, phase information from molecular replacement was sufficient to allow building of the remaining ordered regions. The MsrB3 structure could be built through residue 137 (numbered according to the mature protein sequence as in Fig. 1), but residues 138–156 were initially not visible in the electron density. A truncated version of MsrB3 was then produced, spanning residues 1–137 of the mature protein. This version was crystallized without reductive methylation in a different crystal form, also with two molecules in the asymmetric unit, and diffraction data were collected to 1.71 Å resolution (Table 1). Molecular replacement using the initial MsrB3 model was used to phase the new crystal form. On further inspection of electron density maps from the full-length protein crystals, a segment corresponding to residues 150–156, most likely arising from molecule B in the asymmetric unit, was detected, making interactions with symmetry-related versions of both the A and the B molecules.

**Table 1.** Data Collection and Refinement Statistics

Data collection	Msrb3 (1–156)	Msrb3 (1–137)
Space group	P2_1_2_1_2_1_	P2_1_2_1_2
Unit cell dimensions		
*a*, *b*, *c* (Å)	44.05, 65.30, 112.89	81.15, 85.71, 49.17
*α*, *β*, *γ* (°)	90, 90, 90	90, 90, 90
Resolution (Å)	1.85	1.71
*R*_pim_	0.105	0.041
Mean *I*/*σ*(*I*)	4.9	11.3
Completeness (%)	99.8	99.2
Multiplicity	6.7	4.2
Wilson *B* factor (Å^2^)	16.4	22.15
Refinement		
Resolution (Å)	42.67–1.87	42.65–1.71
No. of reflections/test set	27,651/1383	37,458/1835
*R*_work_/*R*_free_	0.182/0.226	0.174/0.222
No. of atoms^a^		
Protein	2223	2194
Water	325	352
Other	43	16
RMSD from ideal geometry		
Bond lengths (Å)	0.007	0.007
Bond angles (°)	0.899	0.835
Ramachandran		
favored (%)	99.60	98.90
outliers (%)	0	0
Rotamer outliers	0	0.82

^a^ Covalent modifications of protein amino acids, including methyl groups on lysine and the dimethylarsinoyl modification of cysteine, are included in “other.” Also included in “other” are MPD and zinc, sulfate, and chloride ions. Only non-hydrogen atoms were counted.

MPD, 2-methyl-2,4-pentanediol; Msr, methionine sulfoxide reductase; RMSD, root mean square deviation.

**FIG. 2.**
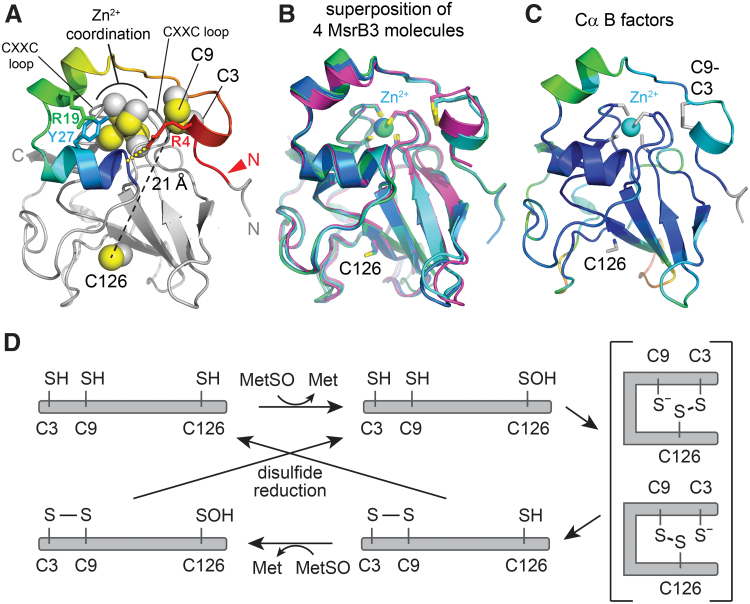
**Structure of human MsrB3. (A)** Ribbon diagram of MsrB3 with cysteine side chains shown as spheres with van der Waals radii. Residues 1–32 are displayed in a rainbow gradient (*red*→*blue*). The termini are labeled in *gray*, with the *red* “N” and *arrowhead* indicating the first amino acid in mature MsrB3. The *dashed black line* indicates the distance between the putative resolving cysteines and the active site. The series of *yellow dots* indicates a C-capping interaction between Arg4 and the carboxy terminus of the *blue helix*. **(B)** Superposition of the four molecules in the asymmetric units of the MsrB3 crystals (truncated version chain A *blue* and chain B *green*; full-length version chain A *magenta* and chain B *cyan*). The zinc ion is shown as a *cyan sphere*, and cysteines are in stick representation. **(C)** MsrB3 (chain B of the truncated form) colored according to *Cα* temperature factors (*blue* low, *orange* high). Cysteines are in stick representation. **(D)** Schematic of the MsrB3 reaction cycle (10). The *bracketed* species are two alternatives for this step in the cycle. Color images are available online.

**FIG. 3.**
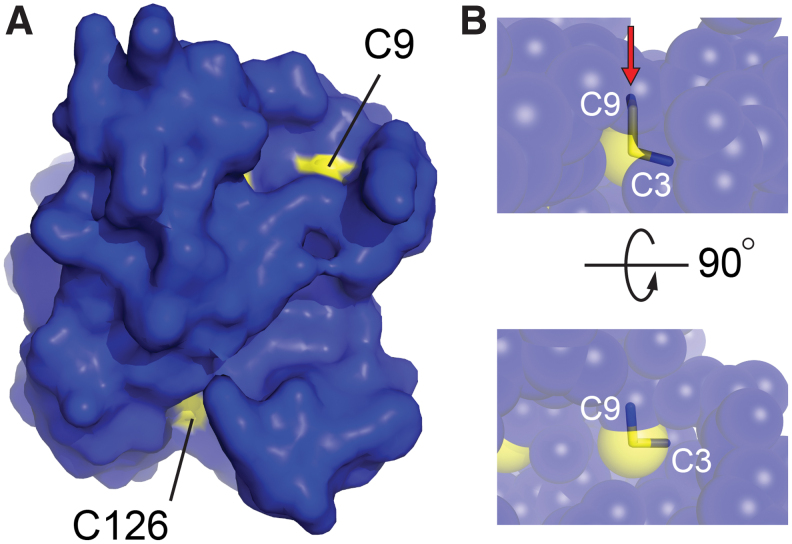
**Accessibility of the CX_5_C disulfide loop. (A)** Surface representation of MsrB3 showing solvent exposure of the active-site cysteine (Cys126) and Cys9 of the Cys3-Cys9 disulfide. **(B)** In a semi-transparent sphere representation, it is evident that Cys9 in the Cys9-Cys3 disulfide (*sticks*) is accessible for nucleophilic attack in the preferred geometry, along the axis of the sulfur-sulfur bond (13), as indicated by the *red arrow* in the *upper panel*. The *lower panel* is oriented such that the nucleophile would approach from above the plane of the page. Color images are available online.

As anticipated, the MsrB3 structure (Fig. 2A) has the characteristic Mss4-like fold (35, 48) found for other MsrB proteins (32), consisting of two highly twisted, short-stranded β sheets. The active-site cysteine, Cys126, is displayed on the outer face of one of the sheets. The structural zinc-binding site found in this fold family (48) is formed in MsrB3 from the four cysteine residues Cys54, Cys57, Cys103, and Cys106, positioned on two opposing loops. The cysteines Cys3 and Cys9, in the CX_5_C motif, were found to be disulfide bonded to one another adjacent to the zinc-coordinating site and far (∼21 Å) from the active-site cysteine, Cys126. The four MsrB3 molecules from the two crystal forms are very similar, including in the CX_5_C region near the amino terminus, with *Cα* root mean square deviation (RMSD) values ranging from 0.5 to 1.0 Å for all pairwise comparisons (Fig. 2B). Certain structural features appear to stabilize the MsrB3 amino-terminal segment, including a cation-π interaction between the two helices that follow the CX_5_C region and the capping of the second helix by Arg4 in the CX_5_C loop (Fig. 2A). Nevertheless, the temperature factors of the first 20 residues are slightly higher than those of the underlying core fold (Fig. 2C). Notably, the CX_5_C cysteines are proposed to be the resolving cysteines that regenerate the MsrB3 active site after its oxidation to sulfenic acid (10) (Fig. 2D). To perform this function, the CX_5_C disulfide would first have to be reduced, and then either Cys3 or Cys9 would need to approach Cys126. Though the amino-terminal region is uniformly packed against the rest of the protein in the MsrB3 crystal structures, the CX_5_C disulfide is exposed to solution (Fig. 3A), and the disulfide is positioned in the correct geometry for an exogenous reductant to perform nucleophilic attack along the Cys9-Cys3 sulfur-sulfur bond (13) (Fig. 3B).

### Comparison with other MsrB structures

**FIG. 4.**
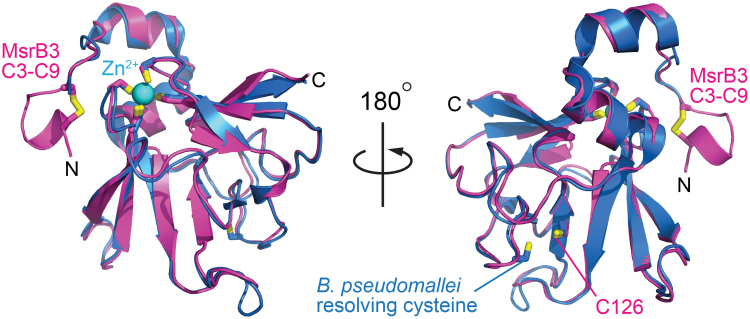
**Comparison of human MsrB3 with a bacterial MsrB homolog.** A superposition of human MsrB3 (*magenta*) and *Burkholderia pseudomallei* MsrB (*blue*; PDB code 3CEZ) is shown in two views. The human and bacterial structures show remarkable similarity, with an RMSD of only 0.8 Å for *Cα* atoms and precise structural alignment of the zinc-binding region (*cyan sphere* in *left* view) and the active-site cysteines (indicated by the C126 label in the view on the *right*). No segment corresponding to the Cys3-Cys9 disulfide of MsrB3, however, is present in the bacterial enzyme, and human MsrB3 lacks a cysteine at the position of the resolving cysteine in *B. pseudomallei*. PDB, protein data bank. Color images are available online.

Aside from the region of the CX_5_C cysteines, human MsrB3 shows a high degree of similarity to bacterial enzymes, represented in the protein structure data bank by *Burkholderia pseudomallei* methionine-R-sulfoxide reductase (PDB code 3CEZ; sequence identity 62% over 119 amino acids) (Fig. 4) and the MsrB domain of *Neisseria gonorrhoeae* PilB (PDB code 1L1D; sequence identity 49% over 135 amino acids). For 129 structurally aligned residues in each case, a *Cα* RMSD of only 0.8 Å was observed between human MsrB3 and each of these crystal structures according to an automated structure comparison against the PDB (17). In contrast, 3.3 and 3.2 Å RMSD values over only 83 and 94 residues, respectively, were measured in pairwise comparisons with the nuclear magnetic resonance (NMR) structures of mammalian MsrB1 (PDB code 2KV1) (2) and MsrB2 (PDB code 2L1U) (1), despite 34% and 46% sequence identity in the aligned regions. The large differences measured between human MsrB3 and other mammalian MsrB enzymes may be due to insufficient restraints for NMR structure determination (1) and deviations from expected geometry in the NMR structure models, as identified by using the Molprobity structure validation tool (11). Notably, the amino-terminal segments of the MsrB1 and MsrB2 structures were particularly poorly resolved (1, 2), in contrast to the CX_5_C region of multiple MsrB3 molecules from the crystal structures, which showed homogeneous structure and packing (Fig. 2B).

**FIG. 5.**
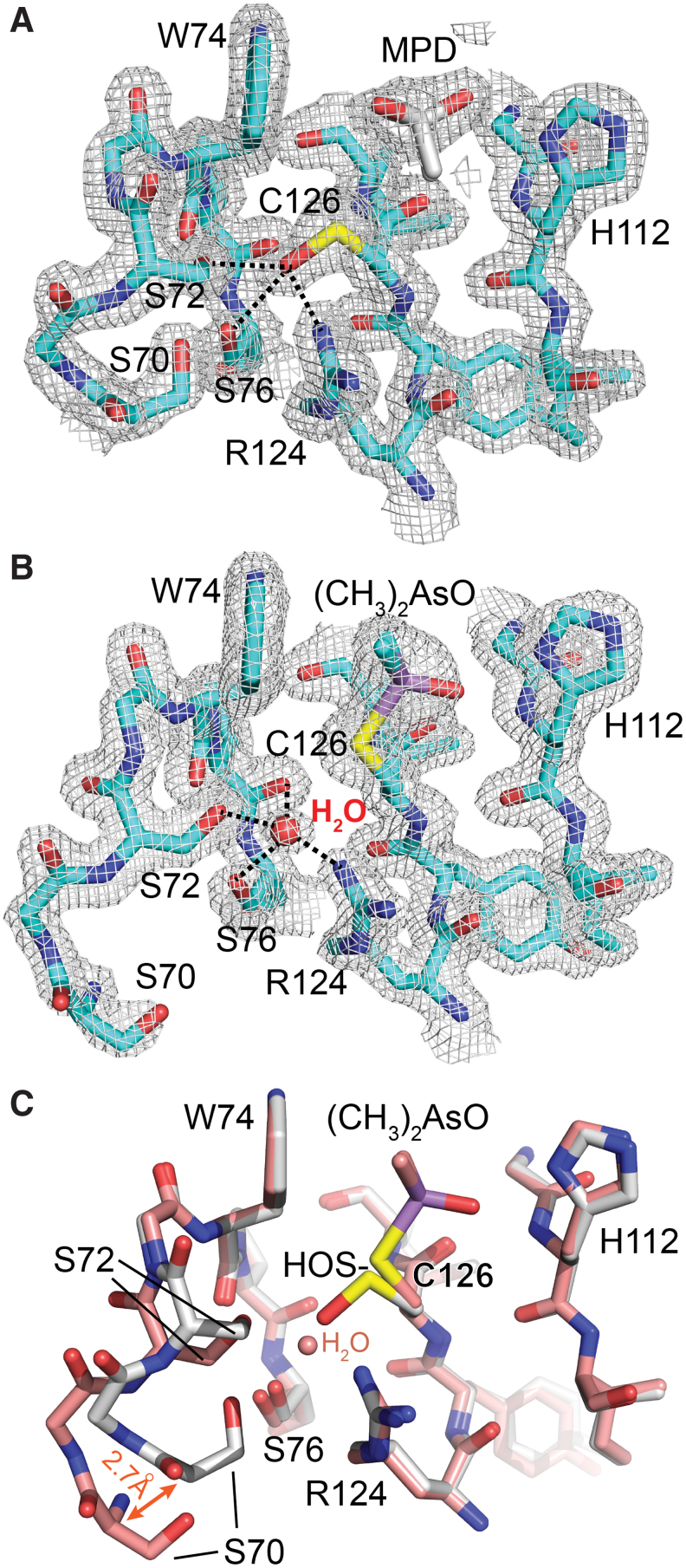
**MsrB3 active-site region in the two crystal forms. (A)** MsrB3 bearing a sulfenic acid in the active site. Hydrogen bonds involving the sulfenic acid are shown by *dashed lines*. **(B)** Dimethylarsinoyl ((CH_3_)_2_AsO) cysteine derivative of MsrB3. Hydrogen bonds to the bound water molecule are indicated. **(C)** Superposition of long (carbon atoms colored *white*) and short (carbon atoms colored *peach*) versions of MsrB3 corresponding to the forms shown in **(A)** and **(B)**, respectively. The different positions of Ser70 in the two structures may be a result of crystal packing and a nearby bound sulfate in the structure of truncated MsrB3. Color images are available online.

A feature shared between MsrB3 and certain homologous structures determined by X-ray crystallography (*e.g.*, PDB code 1L1D) is the presence of a solvent or buffer molecule in the active site, or covalent active-site modifications (Fig. 5). In the structure of the longer version of MsrB3, this species was modeled as 2-methyl-2,4-pentanediol (MPD) (Fig. 5A), which was present during crystallization and was consistent with the appearance of electron density in simulated annealing composite omit maps. In addition, electron density corresponding to a sulfenic acid was seen for the active-site cysteine in this crystal form, with the hydroxyl group oriented toward a conserved pocket rich with hydrogen bond donors and acceptors (Fig. 5A). In particular, the side chain hydroxyls of Ser72 and Ser76 as well as a guanidinium nitrogen of Arg124 are all within about 3 Å of the hydroxyl of the sulfenic acid. Though no substrate was added during the preparation of MsrB3 for crystallization, the sulfenic acid modification may have occurred as a byproduct of formaldehyde addition during the methylation reaction. The fortuitous modification of the active-site cysteine may help shed light on the mechanism for stabilizing the sulfenic acid intermediate and preventing it from engaging in unwanted reactions before it can be cleared by reduced resolving cysteines.

Truncated MsrB3 also contained additional electron density in the active site (Fig. 5B). In this case, the species appeared to be covalently bound to the active-site cysteine. Based on previous observations that crystallization in the presence of cacodylate buffer can result in modified cysteine residues (31, 44), together with inspection of simulated annealing composite omit maps, the density was modeled as a dimethylarsinoyl modification, in which the arsenic is bonded to the cysteine sulfur by displacement of one of the oxygen atoms of the cacodylate. Ser72 and Ser76 coordinate a water molecule instead of a sulfenic acid in the dimethylarsinoyl-modified form. A comparison of the active-site regions of the two crystal forms shows good superposition of all the functional groups that are in hydrogen bonding distance of the sulfenic acid or bound water molecule (Fig. 5C). Only the region around Ser70, which forms hydrogen bonds to Ser72 and Ser76 but is 4.0 Å away from the sulfenic acid, is altered in the dimethylarsinoyl-modified form due to a local and minor change in loop structure with a maximum backbone displacement of 2.7 Å (Fig. 5C).

### The MsrB3 resolving cysteines are oxidized by the active site

Evidence to date indicates that Cys3 and Cys9 function as resolving cysteines in MsrB3 (10). However, these cysteines are more than 21 Å away in Euclidean distance from the active-site Cys126 in the crystal structures presented here (Fig. 2A). Conformational changes have previously been observed in Msr enzymes to facilitate the engagement of the active-site cysteine by a resolving cysteine (40, 41). To examine biochemically whether the CX_5_C cysteines can function together to directly reduce the active site in the absence of another reductant, we reduced these cysteines with dithiothreitol (DTT), removed excess DTT, and supplied the reduced protein with MetSO to oxidize Cys126 to sulfenic acid. This and all subsequent experiments were done with the shorter version of MsrB3, ending at residue 137. After an incubation period, maleimide-functionalized polyethylene glycol (PEG-mal) of molecular weight 5000 Da was added to modify the remaining thiol groups. In this experiment, transfer of electrons from the reduced CX_5_C cysteines to the active site results in a CX_5_C disulfide, which does not react with PEG-mal. In addition, when the active-site Cys126 is in sulfenic acid form it also becomes protected from PEG-mal modification. The number of PEG-mal modifications can be determined by the migration rate of the protein in gel electrophoresis, with cysteine mutants aiding in the assignment of PEG-modified versions of the wild-type protein. For kinetic experiments of this type, electron transfer reactions can be quenched by rapidly lowering the pH, for example by adding trichloroacetic acid. The subsequent PEG-mal modification is carried out after removal of small-molecule oxidizing or reducing agents, when the protein is in the denatured state. This protocol is not suitable for MsrB3, however, as the four zinc-binding cysteines may react with PEG-mal if the zinc is removed by acidification and protein denaturation. Consequently, MsrB3 was treated with PEG-mal in the native state, and then DTT was added to react with remaining PEG-mal and to preserve the zinc-binding cysteines reduced and unmodified for gel electrophoresis.

**FIG. 6.**
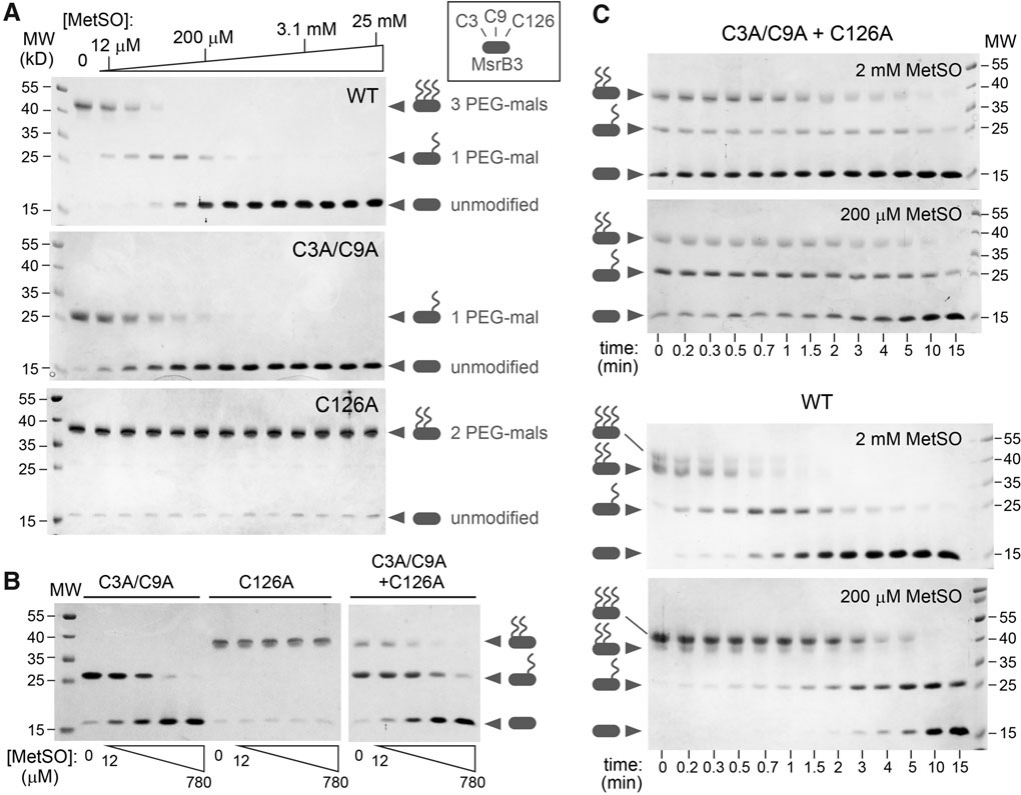
**Addition of MetSO results in oxidation of the CX_5_C cysteines mediated by the active site. (A)** The indicated MsrB3 variants were supplied to a set of parallel reactions with the CX_5_C and active-site cysteines (when present) reduced. MetSO was added from a series of twofold dilutions. Some of the MetSO concentrations in the reactions are labeled on the wedge above the gels. After a 15-min incubation, PEG-mal was added to modify the remaining free thiols. Resistance to PEG-mal modification at increasing MetSO concentrations was a result of disulfide formation by the CX_5_C cysteines and sulfenic acid formation at the active site. The Cys126Ala active-site mutant showed no change with MetSO addition, indicating that Cys126 normally catalyzes oxidation of the Cys3-Cys9 disulfide in the presence of MetSO. **(B)** Electron transfer between the resolving cysteines and the active site can occur intermolecularly. Cys126Ala is not oxidized in 15 min when treated with MetSO, but it becomes oxidized and resistant to PEG-mal modification when Cys3Ala/Cys9Ala, which contains a functional active site, is also present. MetSO was supplied as a series of fourfold dilutions. **(C)** A kinetic analysis showed that protection of Cys3 and Cys9 from PEG-mal modification in the Cys126A mutant by mixing with the Cys3A/Cys9A mutant occurred slowly at two MetSO concentrations. In contrast, disulfide formation and protection from modification occurred rapidly in the wild-type enzyme at a higher MetSO concentration. At the higher concentration, MetSO competed with PEG-mal for the active site, such that the zero and early time points show some degree of protection of Cys126 from PEG-mal. Together, these experiments show that oxidation of the CX_5_C cysteines is more efficient by intra- than intermolecular electron transfer and that intramolecular transfer is limited by the rate of active-site modification in the range of MetSO concentrations studied. MetSO, methionine sulfoxide; PEG, polyethylene glycol.

The first experiments to monitor electron transfer from the CX_5_C cysteines to the active site were conducted at a fixed incubation time. With increasing MetSO concentrations, MsrB3 became resistant to PEG-mal modification during the incubation, corresponding to protection of three cysteines (Cys3, Cys9, and Cys126) (Fig. 6A). The Cys3Ala/Cys9Ala mutant also became resistant to PEG-mal modification on MetSO addition, due to sulfenic acid formation at Cys126. In contrast, the Cys126Ala mutant did not show any change in migration on addition of MetSO, remaining sensitive to PEG-mal modification at Cys3 and Cys9 across the whole MetSO concentration range, indicating that oxidation of the CX_5_C cysteines depends critically on a functional active site. Comparing wild-type MsrB3 with the Cys3Ala/Cys9Ala mutant, it can be seen that protection of the mutant from PEG-mal modification occurred at lower MetSO concentrations (Fig. 6A). Low MetSO concentrations resulted in disappearance of fully reduced wild-type MsrB3, but a species with a single PEG-mal modification remained. This observation likely reflects the fact that the reduced wild-type protein can undergo two rounds of sulfenic acid formation at Cys126 when MetSO is added (Fig. 2D). The first sulfenic acid is restored to thiol form by the reduced CX_5_C cysteines, resulting in a CX_5_C disulfide and PEG-mal reactivity at only Cys126. If the active site is oxidized a second time, there are no remaining CX_5_C thiols to reduce it, and the enzyme becomes protected from PEG-mal modification at all three redox-active cysteines.

In principle, oxidation of the CX_5_C cysteines could occur intermolecularly. To test this possibility, the Cys3Ala/Cys9Ala and Cys126Ala mutants were mixed in the reaction, and the products were compared with those of each mutant alone. Under these circumstances, the Cys126Ala mutant became resistant to PEG-mal modification (Fig. 6B), indicating that intermolecular electron transfer from the resolving cysteines in one enzyme molecule to the active site of another enzyme molecule is a viable electron transfer route for MsrB3. However, comparing the kinetics of oxidation of wild-type MsrB3 with the kinetics of the mixture of the two complementary mutants demonstrated that the intramolecular electron transfer route is preferred when available (Fig. 6C). MetSO modification of the active site is rate limiting for wild-type MsrB3, such that protection of the redox-active cysteines from PEG-mal modification was strongly dependent on MetSO concentration. In contrast, the mixture of cysteine mutants showed a weak dependence on MetSO concentration, suggesting that intermolecular electron transfer was rate limiting in this case. We conclude that intramolecular electron transfer efficiently regenerates a reduced MsrB3 active site and produces an oxidized CX_5_C motif, though intermolecular electron transfer occurs to some extent when the intramolecular route is unavailable.

### Analysis of a stabilized intermediate with a Cys9-Cys126 disulfide

**FIG. 7.**
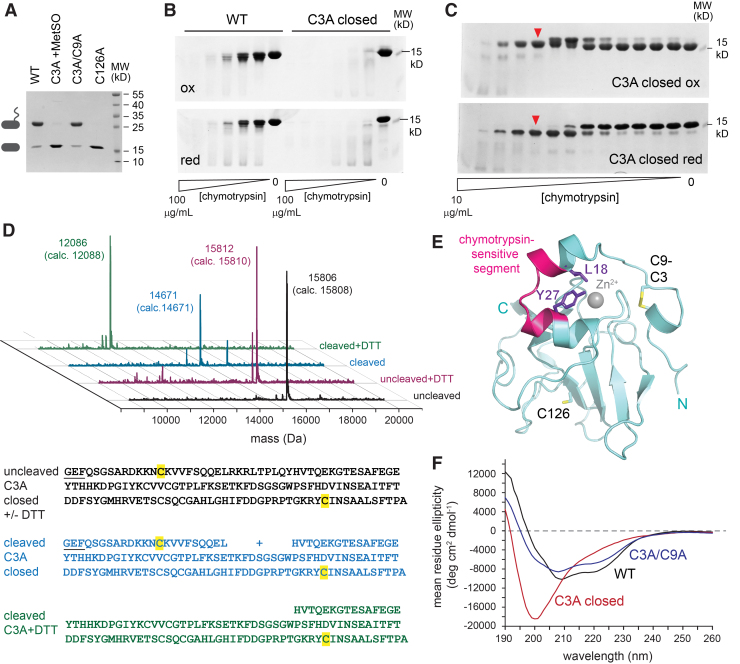
**Internal disulfide bond and increased protease sensitivity in the Cys3Ala intermediate-state mimic. (A)** Treatment of the Cys3Ala mutant with MetSO produced a species that is nonreactive with PEG-mal. Shown for comparison are untreated wild-type MsrB3 and the Cys3Ala/Cys9Ala mutants, which receive one PEG-mal modification at Cys126. The Cys126Ala mutant is resistant to modification, because the remaining redox-active cysteines are protected in a Cys3-Cys9 disulfide. **(B)** MetSO-treated Cys3Ala is more sensitive than wild-type MsrB3 to proteolysis with chymotrypsin. Wedges represent a range of chymotrypsin concentrations. Samples from the digestions were applied to gels in the oxidized (ox) form or after reduction (red) with DTT. **(C)** limited proteolysis produced a species that migrated more slowly than the uncleaved protein under oxidizing (C3A closed ox) conditions. Under reducing conditions (C3A closed red), a fragment that migrated faster than the full-length protein was liberated. A sample prepared as for the lanes indicated by *red arrowheads* was applied to LC-MS. **(D)** LC-MS results for the species indicated in panel C and for uncleaved controls. Measured masses are shown for the major peak in each sample. In *parentheses* are the calculated masses for each species, the amino acid sequences of which are shown below the spectra. Amino acids remaining from the cloning site and TEV cleavage of the fusion protein, and that are not part of MsrB3, are *underlined*. Cysteine amino acids are highlighted in *yellow*. The “cleaved” sample was assigned as a species missing residues 19–27 but containing amino-terminal residues linked by a disulfide to the carboxy-terminal region. The “cleaved + DTT” sample was assigned as residues 28–137, with the latter being the carboxy terminus of the short MsrB3 variant. **(E)** The chymotrypsin-sensitive segment of oxidized Cys3Ala is shown in the context of the structure of wild-type MsrB3, in which this segment is folded and protected. The conformation of oxidized Cys3Ala is expected to be different from the structure shown, because it contains a disulfide between Cys9 and Cys126. Further, proteolytic cleavage of the Cys3Ala intermediate-state mimic occurred at the peptide bonds after Leu18 and Tyr27, which are not exposed in the wild-type structure. **(F)** Circular dichroism spectra of wild-type MsrB3, the Cys3Ala/Cys9Ala mutant, and the oxidized Cys3Ala intermediate-state mimic. Oxidized Cys3Ala shows loss of helical signal (minima at 222 and 208 nm). DTT, dithiothreitol; LC-MS, liquid chromatography mass spectrometry; TEV, tobacco etch virus. Color images are available online.

Reduction of the MsrB3 active site by the CX_5_C cysteines requires a disulfide intermediate between Cys3 or Cys9 and Cys126 (Fig. 2D). Nucleophilic attack on this intermediate by the partner CX_5_C cysteine would restore the Cys126 thiol and form a Cys3-Cys9 disulfide, suggesting that mutation of the attacking cysteine would stabilize the intermediate. We generated the Cys3Ala and Cys9Ala mutants and observed that both yielded a substantial fraction of oxidized monomeric species on treatment with MetSO. The monomeric, oxidized Cys3Ala mutant (Fig. 7A) was purified from minor intermolecular disulfide contaminants by gel filtration. Monomeric, oxidized Cys3Ala was designated the “closed” form of this mutant, because a putative intramolecular disulfide sealed off its active site. Despite obtaining high yields of homogenous closed Cys3Ala MsrB3 that could be concentrated to at least 20 mg/mL, numerous attempts to crystallize this variant, prepared with or without reductive methylation, were unsuccessful. One explanation may be increased flexibility of the oxidized Cys3Ala, which was found to be much more sensitive than wild-type MsrB3 to proteolytic digestion using chymotrypsin (Fig. 7B).

Optimized digestion of oxidized Cys3Ala using lower chymotrypsin concentrations revealed a preferred protease-sensitive site. Cleavage slowed the migration of the product in the oxidized state, a phenomenon likely due to an effect on the hydrodynamic properties of the protein rather than to an increase in size. In the reduced state, a major band that migrated more rapidly than the intact protein was detected, consistent with liberation of a disulfide bonded cleavage product (Fig. 7C). The intact and chymotrypsin-clipped Cys3Ala were subjected to liquid chromatography mass spectrometry (LC-MS) (Fig. 7D). Chymotrypsin cleavage produced a mass consistent with a species containing both termini of the protein but missing nine amino acids (residues 19 through 27) from the helical region downstream of the CX_5_C motif (Fig. 7E). Reduction of the cleaved protein produced the expected mass for the fragment spanning residue 28 to the carboxy terminus (Fig. 7D), indicating that the amino terminal region had, indeed, been linked to the carboxy terminal fragment by a disulfide bond. The chymotrypsin recognition sites, Leu18 and Tyr27, are packed against one another and buried in the wild-type MsrB3 structure (Fig. 7E), but their protease sensitivity in the Cys3Ala intermediate-state mimic suggests that disulfide formation between Cys9 and Cys126 is facilitated by disruption of the helical region. Indeed, circular dichroism spectroscopy of oxidized Cys3Ala lacked the helical signal, a double minimum at 222 and 208 nm, exhibited by wild-type MsrB3 (Fig. 7F). The CX_5_C disulfide is not required to stabilize the MsrB3 helices, as the Cys3Ala/Cys9Ala mutant shows a helical signal similar to the wild-type enzyme (Fig. 7F). Considering the circular dichroism and chymotrypsin cleavage results together, the helical, amino-terminal region of oxidized Cys3Ala is structurally reorganized, but other parts of the MsrB3 protein appear to remain folded, as judged by the high selectivity of chymotrypsin cleavage despite the numerous possible protease recognition sites.

## Discussion

The major finding from the human MsrB3 crystal structure is that the resolving cysteines, Cys3 and Cys9, form a disulfide bond and are distant from the active site in the observed conformation of the enzyme. This result could not readily have been predicted, as the MsrB3 resolving cysteines are outside the region of homology with known structures of other MsrB proteins. In many bacterial MsrB enzymes, resolving cysteines are adjacent in space to the active-site cysteines within the core β-sheet-rich region of the fold (32). The mammalian MsrB proteins, however, have diverged from this paradigm and have a serine or threonine in place of the proximal resolving cysteine (22). Instead, the resolving cysteines of mammalian enzymes are located in different regions of their primary structures, and as the MsrB3 structure reveals, in different regions of the tertiary structure.

Analyzing previous biochemical experiments in light of the MsrB3 structure now provides insights into the catalytic mechanism. In assays with purified enzyme, mutation of either Cys3 or Cys9 compromised regeneration of the MsrB3 active site by thioredoxin (10, 22), whereas mutation of both cysteines together dramatically decreased recycling (10). These results demonstrate that thioredoxin does not directly reduce the sulfenic acid at the MsrB3 active site. Further, the partial activity seen for the single mutants suggests that either of the CX_5_C cysteines can initiate mixed disulfide formation to expel the oxygen at the active-site cysteine sulfur. Nevertheless, the most effective version of the enzyme was wild-type MsrB3 containing both Cys3 and Cys9. The observation of a Cys3-Cys9 disulfide in the crystal structure suggests that, after a mixed disulfide has formed between one of the CX_5_C cysteines and the active-site cysteine, the other CX_5_C cysteine reduces the mixed disulfide, regenerating the active-site thiol and yielding the Cys3-Cys9 disulfide. Consistent with this notion is the observation that exposure of reduced MsrB3 to MetSO results in disulfide bond formation between Cys3 and Cys9 (Fig. 6).

The MsrB3 crystal structure further revealed that Cys9 is exposed to solvent and is, thus, the likely target for nucleophilic attack to reduce the Cys3-Cys9 disulfide and drive a further enzymatic cycle. The existence of a double-displacement mechanism, involving two successive thiol-disulfide rearrangements, has been described for *E. coli* MsrA (8), but in that case the two resolving cysteines are located near the carboxy terminus of the enzyme and are not disulfide bonded to one another in the structures that have been determined (44), thus differing from MsrB3.

While addressing certain mechanistic issues, the MsrB3 structure raises the questions as to how the distal cysteines interact with the MsrB3 active site and what evolutionary advantages arise from moving the resolving cysteines away from the active-site region (22). One possible answer is that the pair of resolving cysteines in MsrB3 may function in an analogous manner to the “shuttle” disulfides in other ER-localized enzymes that transfer electrons by dithiol-disulfide exchange. The direct electron donor for MsrB3 recycling in its natural setting may be a member of the PDI family, as PDIs catalyze various thiol-mediated redox reactions in the ER such as formation of disulfides in secreted proteins, reduction of disulfides in proteins destined for degradation, and the reversible regulation of enzyme activity. In a number of cases, PDI-family oxidoreductases interact with shuttle disulfides instead of directly with core enzyme active sites. One example is Ero1, an ER sulfhydryl oxidase that generates disulfides by transferring electrons from cysteines to hydrogen peroxide (16). The Ero1 catalytic site consists of a disulfide in a CXXC motif adjacent to a bound flavin adenine dinucleotide cofactor (15). PDI family oxidoreductases do not directly access this active site, however, but rather reduce a CX_4_C disulfide (14) on an exposed flexible loop of Ero1 (15). The resulting reduced cysteines in the loop then reduce the Ero1 FAD-proximal disulfide, effectively shuttling electrons from PDI to the Ero1 active site. Another example of a shuttle disulfide in the ER occurs in the transmembrane protein VKOR (29). The VKOR CX_7_C shuttle disulfide transfers electrons from membrane-bound PDI family proteins to the VKOR active-site disulfide next to the quinone cofactor, from where they can be used to reduce vitamin K epoxide (43). It is believed that, in general, shuttle disulfides facilitate the function of redox enzymes by enabling substrate specificity to evolve independently of core catalytic activity (13), and MsrB3 may be another example of this phenomenon.

Ero1, VKOR, and MsrB3 all appear to accept electrons from reduced cysteine thiolates *via* their shuttle disulfides. This mechanistic similarity suggests that one potential source of reducing equivalents for re-activating MsrB3 in the ER would be cysteines in nascent polypeptide chains, which might transfer electrons *via* PDI family proteins to MsrB3. Other sources of reducing equivalents in the ER are also possible, including reduced glutathione (25). For comparison, recycling of MsrB enzymes in the bacterial periplasm, an environment analogous to the ER in its support of oxidative protein folding, involves electrons transferred across the cell membrane by the enzyme DsbD (7). It should be noted, however, that oxidized MsrB3 can convert free methionine to the sulfoxide (10), indicating that an environment that favors conversion of the MsrB3 active site to sulfenic acid will, in turn, drive formation of MetSO. Whether the reaction runs in this direction *in vivo* in certain circumstances remains to be determined.

Regardless of the direction of electron transfer, a key step in the MsrB3 mechanism requires the CX_5_C cysteines to approach the active-site cysteine, for which a major conformational change is required. The MsrB3 crystal structure shows that the amino-terminal region containing the two helices is ordered and exhibits the same conformation in all four molecules in the two crystal asymmetric units in this study. The similarity between the organization of the two helices in MsrB3 and in the *B. pseudomallei* MsrB (Fig. 4) further suggests that this arrangement is not fortuitous but rather is a stable, evolved feature. Nevertheless, some movement of at least the amino-terminal of the two helices is likely required to enable the CX_5_C cysteines to reach the active site, as the segment of five amino acids between Cys9 and the start of the first helix is insufficient to span the required distance (Fig. 2A). Crystal structures of the *Xanthomonas campestris* MsrB enzyme show how a polar pocket corresponding to the one that accommodates the sulfenic acid in MsrB3 is disrupted by a change in conformation to enable formation of a disulfide between the resolving and active-site cysteines in that enzyme (41). However, the resolving cysteine is downstream of the amino-terminal helices in *X. campestris* MsrB but upstream of the helices in animal MsrB3. Therefore, it is not yet clear whether the resolution of the active site in MsrB3 will similarly involve disruption of the polar pocket. In this study, we detected changes in the conformation and accessibility of the amino-terminal region of the protein that likely facilitate approach of the resolving cysteines toward the enzyme active site. Now that the scale of the structural rearrangements required to resolve the sulfenic acid intermediate is known, how these rearrangements occur in MsrB3 and how ER factors engage the resolving cysteines to promote MsrB3 recycling can begin to be investigated.

## Materials and Methods

### Protein production and purification

MsrB3 was produced as a fusion protein downstream of MBP, a His_6_ tag, and a TEV protease cleavage site. Cleavage at the TEV site resulted in the following amino-terminal sequence: GEFQSGSC…, in which the underlined amino acids arise from an EcoR1 restriction site. Residue 1 in the MsrB3 structure corresponds to the first amino acid of the mature sequence according to UniProt (residue 33 of the MsrB3 precursor sequence) (45). The original expression construct extended through residue 156 of the mature protein, that is, lacking only the KAEL sequence at the carboxy terminus. After the MsrB3 structure was solved by using this construct, a second plasmid was generated by introduction of a stop codon after residue 137. Plasmids were transformed into the BL21(DE3) *E. coli* strain. Cultures were grown at 37°C in the presence of 100 mg/L ampicillin to an optical density of 0.5 at 595 nm, at which point isopropyl β-D-1-thiogalactopyranoside and zinc chloride were added, both at a concentration of 0.5 mM. The growth temperature was lowered to 25°C, and the cultures were left to shake overnight. Cells were then pelleted, resuspended in 5 mM sodium phosphate, pH 7.5, 125 mM NaCl, and 5 mM imidazole, and frozen at −80°C.

To purify the MsrB3 protein from bacterial cells, cell suspensions were thawed, and the protease inhibitors phenylmethylsulfonyl fluoride, leupeptin, and pepstatin were added. The cell suspensions were then sonicated on ice and spun at 25,000 g for 20 min at 4°C. Supernatant was applied to a Ni-NTA column, washed in the cell suspension buffer, and finally eluted with an increasing imidazole gradient. Eluted protein was placed into a dialysis bag together with His_6_-tagged TEV protease and dialyzed against phosphate buffered saline (PBS) overnight at room temperature. The cleaved protein was reapplied to a Ni-NTA column, and MsrB3 was collected from the unbound, flow-through fraction. The protein was exchanged by using a PD-10 column (GE Healthcare no. 17-0851-01) into 25 mM 4-(2-hydroxyethyl)-1-piperazineethanesulfonic acid (HEPES) buffer, pH 7.5, 250 mM NaCl, and the original, longer version was treated as described to methylate lysines (46). The methylated protein was then applied to a gel filtration column in the same buffer. The eluted protein was diluted to 10 mM HEPES, pH 7.5, 100 mM NaCl and concentrated to 15 mg/mL (∼1 mM). The shorter MsrB3 protein was not methylated but was otherwise prepared in the same manner.

### Crystallization and structure solution

The longer version of MsrB3 was crystallized by the hanging drop vapor diffusion method by mixing 1:1 protein stock with 1.6 M ammonium sulfate, 100 mM sodium cacodylate, pH 7.4, and 200 mM sodium chloride at 20°C. Crystals consistently grew as showers of small needles. In one instance, seeding from these crystals into a hanging drop 12 h after mixing 1:1 with well solution containing the original crystallization solution plus 10% MPD produced larger, rod-shaped crystals. Diffraction data were collected from these and other MsrB3 crystals at beamline ID30B of the European Radiation Synchrotron Facility. Molecular replacement was done by using Phenix (3) with residues 10–139 of structure 3CEZ as the search model. Rebuilding was performed by using Coot (12), and refinement was done by using Phenix. The 150–156 fragment was assigned to molecule B based on a gap of only 11 Å between residue 137 of molecule B and residue 150 of the fragment, but contiguous electron density linking residues 137 and 150 was not visible.

The shorter version of MsrB3 crystallized in ∼1.5 M ammonium sulfate, 100 mM sodium chloride, 50 mM cacodylate buffer, pH 6.8, and 15% glycerol. Phases were obtained by molecular replacement with a partially refined structure of MsrB3 obtained as described earlier.

The quality of the MsrB3 structures was validated by Molprobity and by close inspection of composite simulated annealing omit maps calculated by using Phenix. The models of the longer and shorter versions have no residues in disallowed regions of Ramachandran space. The longer form has 99.6% of residues in favored regions, whereas the truncated form has 98.9% of residues in favored regions.

### MetSO assays

MsrB3 variants (short form) were reduced by incubating 200 μL of 100 μM enzyme with 20 mM DTT for 30 min at room temperature. DTT was then removed by buffer exchange using a PD-10 column equilibrated with PBS, collecting elution fractions of 0.5 mL. Protein concentrations were measured after elution, and reduced proteins were diluted to 10 μM in PBS. L-MetSO (Sigma no. M1126) was dissolved at 100 mM in PBS, and dilution series were prepared. Reduced MsrB3 variants were mixed 1:1 with the L-MetSO dilutions and incubated for 15 min at 37°C. During the incubation, 50 mg PEG-mal (5000 Da) (Iris Biotech GmbH no. PEG1149) was dissolved in 200 μL water, yielding a solution of ∼50 mM, and applied to a PD-10 column equilibrated in PBS to remove any unconjugated maleimide. Elution fractions of 0.5 mL were collected, and PEG-maleimide concentration was measured. To do so, a sample taken from the fraction that was faintly yellow in color was diluted 1:10 in PBS, and 20 μL of the dilution was mixed with DTT such that the final volume was 900 μL and the DTT concentration was 11.1 μM. After 5 min, 100 μL from a solution of 2 mM Ellman's reagent [5,5′-dithiobis-(2-nitrobenzoic acid)] (TCI no. D0944) dissolved in PBS was added, and the absorbance was measured. The decrease in absorbance was taken to indicate the concentration of DTT thiols alkylated by PEG-mal, and the concentration of the PEG-mal fraction from the PD-10 column was determined to be about 5 mM. A tenth of the reaction volume of PEG-mal was added to each MsrB3 sample at the end of the incubation with MetSO. After a further incubation for 15 min at room temperature, DTT was added to a concentration of 50 mM. Five μL of each reaction was removed, 5 μL gel loading buffer was added, and the samples were loaded onto a 12% gel. To analyze the mixture of Cys3Ala/Cys9Ala and Cys126Ala, samples were prepared similarly except that proteins were diluted to 20 μM after removal of DTT by using the PD-10 column, and 1:1:2 mixtures of protein:PBS:MetSO or protein:protein:MetSO were prepared for incubation at 37°C.

For kinetic experiments, reactions were performed at 37°C in a volume of 8 μL and initiated by addition of MetSO. At the indicated time points, samples were removed from 37°C, and a tenth of the reaction volume of PEG-mal was added at room temperature. It should be noted that PEG-mal reacts rapidly but not instantaneously with MsrB3 cysteines, such that the early time points reflect a competition between PEG-mal alkylation of all remaining cysteine thiols and MetSO sulfenylation of the active site. Half an hour after the final PEG-mal addition of the time-course, 1 μL of 1 M DTT and 5 μL gel loading buffer were added to each tube. Samples were applied to a 12% polyacrylamide gel.

### Limited proteolysis

The shorter version of wild-type MsrB3 and the oxidized Cys3Ala variant were incubated at 37°C for 1 h at a concentration of 45 μM in PBS with various concentrations of TLCK-treated chymotrypsin (Chem-Impex International, Inc. no. 01783). The highest chymotrypsin concentration was 100 μg/mL for Figure 7B and 10 μg/mL for Figure 7C, and successive lanes represent twofold dilutions of protease. Cleavage was stopped by the addition of phenylmethylsulfonyl fluoride. Gel loading buffer containing either 20 mM N-ethyl maleimide or 20 mM DTT was then added, and samples were applied to 15% polyacrylamide gels.

### Mass spectrometry

Uncleaved or chymotrypsin-cleaved Cys3Ala was applied to LC-MS with or without prior reduction by using DTT. LC-MS runs were performed on a Waters ACUITY UPLC class H instrument, in the positive ion mode by using electrospray ionization. UPLC separation used a C4 column (300 Å, 1.7 μm, 21 × 100 mm). The column was held at 40°C, and the autosampler was held at 10°C. Mobile solution A was 0.1% formic acid in water, and mobile phase B was 0.1% formic acid in acetonitrile. The run flow was 0.4 mL/min with gradient 20% B for 2 min, increasing linearly to 60% B for 3 min, holding at 60% B for 1.5 min, changing to 0% B in 0.1 min, and holding at 0% for 1.4 min. Mass data were collected in the range of 700–1550 m/z on a Waters SQD2 detector. Deconvolution was performed by using the MassLynx program with MaxEnt processing in the range of 9000–20,000 Da. Calculations of expected masses were based on monoisotopic mass and included oxidation of the single methionine in the sequence. The loss of two protons for a disulfide bond in the oxidized samples was taken into account.

### Circular dichroism spectroscopy

Proteins were diluted 100-fold from 1 mM stocks (prepared as for crystallization) into 10 mM sodium phosphate buffer, pH 7.4. Data were collected at 24°C on a Chirascan circular dichroism spectrophotometer (Applied Photophysics) in the automated signal acquisition mode and presented as an average of five repeats.

## Accession Numbers

The coordinates and structure factors have been deposited in the PDB with accession codes 6Q9V and 6QA0 for the longer (sulfenic acid) and shorter (dimethylarsinoly cysteine) MsrB3 constructs, respectively.

## Abbreviations Used

DTT: dithiothreitol
ER: endoplasmic reticulum
HEPES: 4-(2-hydroxyethyl)-1-piperazineethanesulfonic acid
KAEL: Lys-Ala-Glu-Leu
KDEL: Lys-Asp-Glu-Leu
LC-MS: liquid chromatography mass spectrometry
MBP: maltose binding protein
MetSO: methionine sulfoxide
MPD: 2-methyl-2,4-pentanediol
Msr: methionine sulfoxide reductase
NMR: nuclear magnetic resonance
PBS: phosphate buffered saline
PDB: protein data bank
PDI: protein disulfide isomerase
PEG-mal: maleimide-functionalized polyethylene glycol
RMSD: root mean square deviation
TEV: tobacco etch virus
